# Effect of Previous Frozen Storage and Coating Medium on the Essential Macroelement and Trace Element Content of Canned Mackerel

**DOI:** 10.3390/foods12122289

**Published:** 2023-06-06

**Authors:** Ricardo Prego, Antonio Cobelo-García, Beatriz Martínez, Santiago P. Aubourg

**Affiliations:** 1 Department of Oceanography, Marine Research Institute (CSIC), 36208 Vigo, Spain; prego@iim.csic.es (R.P.); acobelo@iim.csic.es (A.C.-G.); 2 Department of Food Technologies, CIFP Coroso, Avenida da Coruña, 174, 15960 Ribeira, Spain; bmartinezr@edu.xunta.gal; 3 Department of Food Technology, Marine Research Institute (CSIC), c/Eduardo Cabello, 6, 36208 Vigo, Spain

**Keywords:** Atlantic mackerel, macroelements, trace elements, frozen storage, coating medium, canning, protein denaturation, liquor loss, lipid changes, nutritional value

## Abstract

The effect of previous frozen storage (−18 °C for 6 months) and different coating media (aqueous: water and brine; oily: sunflower, refined olive, and extra-virgin olive oils) on the essential macroelement and trace element content of canned Atlantic mackerel (*Scomber scombrus*) was studied. Previous frozen storage led to an increased (*p* < 0.05) content of canned samples of *K* (oil-coated samples) and *Ca* (all coating conditions) and to a decreased (*p* < 0.05) content of *P* (aqueous-coating samples) and *S* (water- and oil-coated samples). For trace elements, a content increase (*p* < 0.05) in *Cu* and *Se* (brine-canned samples) and *Mn* (water- and refined-olive-oil-coated samples) was detected in canned fish muscle with frozen storage. Concerning the coating effect, aqueous-coating samples showed lower (*p* < 0.05) *Mg*, *P*, *S*, *K*, and *Ca* contents than their corresponding oil-coated samples. For trace elements, lower average contents were found for *Co*, *Cu*, *Mn*, *Se*, and *Fe* in aqueous-coating fish muscle when compared to their counterparts coated in oily media. Content changes in the different elements in canned fish muscle are discussed based on interactions with other tissue constituents and modifications that such constituents undergo during processing (i.e., protein denaturation, liquor losses from the muscle, lipid changes).

## 1. Introduction

The majority of the macroelements and trace elements considered essential for human biological processes, including growth, antioxidant defence, reproduction, and hormone metabolism, are present in marine fish and invertebrates [1,2,3]. Marine species can accumulate minerals from the diet and deposit them in their skeletal tissues and organs. Remarkably, the concentration of elements in seafood may be influenced by different factors such as nourishment source, environment, species, season, tissue, maturation degree, and processing [4,5,6].

Canning is a traditional process widely employed for marine species preservation [7,8]. The nature of the raw material can be altered substantially by the heat treatment involved to produce a food product with different characteristics [9,10]. Both bacteria and endogenous enzymes should be permanently inactivated by heat treatment and lead to a safe and durable food, provided that further contamination does not occur. Different kinds of coating media (namely, aqueous and oily) can be employed for canned fish commercialisation, all of them receiving great acceptance from consumers [11]. However, marine species are reported to be highly sensitive to heat processing, which leads to heat degradation, toughening and drying of fish muscle, oxidation of constituents, and leaching of water-soluble constituents [12,13,14]. With regards to the mineral content of canned fish muscle, the most important events would be protein denaturation, minerals being released from the fish tissue, and resulting liquor losses, including essential macroelements and trace elements from the canned muscle into the coating medium [15,16,17].

On the basis of canneries’ needs for raw material availability, frozen storage has been the most employed condition for prior preservative storage. However, deterioration during the frozen storage of fish is susceptible to continuing (lipid oxidation, hydrolysis development, and protein denaturation) as a result of endogenous enzyme activity, especially if convenient storage temperatures are not respected and long-term storage periods are needed [18,19,20]. Furthermore, the resulting quality changes during the frozen storage period may have relevant detrimental effects on the quality of the canned product [13,21,22].

This study focused on the essential macroelement (*Ca*, *K*, *Mg*, *Na*, *P*, and *S*) and trace element (*Co*, *Cu*, *Fe*, *Mn*, and *Se*) contents of canned Atlantic mackerel (*Scomber scombrus*). The objective was to analyse the effect of a previous 6-month storage at −18 °C and the employment of different kinds of coating media (aqueous: water and brine; oily: sunflower, refined olive, and extra-virgin olive oils) on the essential element content of canned muscle. Additionally, comparison of element contents between initial raw fish and canned samples was also undertaken.

## 2. Materials and Methods

### 2.1. Initial Fish and Frozen Storage

Fresh Atlantic mackerel (*S. scombrus*) (110 specimens; weight and length ranges: 215–255 g and 27.5–31.0 cm, respectively) were obtained at Vigo harbour (Galicia, Spain) in November 2020. Once in laboratory, ten specimens were taken, divided into five batches (two specimens per batch), beheaded, eviscerated, and filleted. Then, the white muscle was separated from fillets, pooled together within each batch, minced, and employed to carry out the different element analyses of the initial fish (*n* = 5).

Meanwhile, 50 specimens were taken and divided into 5 batches (10 specimens per batch). The fish were beheaded, eviscerated, filleted, and subjected to the canning procedure (samples without prior freezing and frozen storage; Group I). The remaining fish (50 specimens) were stored at −40 °C for 48 h (freezing step) and then kept frozen (−18 °C) for 6 months. After this time, specimens were thawed overnight (4 °C) and divided into five batches (ten specimens per batch). Fish were beheaded, eviscerated, filleted and subjected to the canning procedure (samples with prior freeing and frozen storage; Group II).

Freezing and frozen storage conditions (i.e., −40 °C for 48 h and −18 °C for 6 months, respectively) were chosen according to common practices employed in canneries.

### 2.2. Canning Process

At each canning time, 45 g portions of mackerel fillets were placed in flat rectangular cans (105 × 60 × 25 mm; 150 mL). As coating media, water, brine (aq. 2% NaCl solution), sunflower oil (SFO), refined olive oil (ROO), and extra-virgin olive oil (EVOO) were employed. Cans were fulfilled with the corresponding coating media. A single fish was employed for the preparation of each can.

After vacuum sealing, cans were subjected to the sterilisation process in a horizontal steam retort (115 °C, 45 min; *F*_o_ = 7 min) (CIFP Coroso, Ribeira, A Coruña, Spain) [23]. After completing the heating time, steam was cut off, and the remaining steam was flushed away with air. Then, the cans were stored at room temperature (20 °C) for 3 months. A minimum of a 2-month time period is considered necessary in canneries in order to optimise fish palatability in commercial canned fish [24].

### 2.3. Sampling Procedure of Canned Samples

After the canned storage, both kinds of canned samples (canned Groups I and II) were opened, and the liquid part was carefully drained off gravimetrically. Then, the fish muscle was separated, and the remaining coating medium was eliminated from the fish tissue by wrapping with filter paper.

The fish white muscle of two cans with the same coating medium was pooled together, minced, and employed for the different analyses. Cans corresponding to each coating medium were analysed by means of five replicates (*n* = 5).

### 2.4. Determination of Moisture Content

Moisture was determined as the weight difference (1–2 g) before and after 4 h at 105 °C in agreement with the official method, 950.46B [25]. Results were calculated as g·kg^−1^.

### 2.5. Determination of Element Presence in Initial Fish and Canned Samples

Contents of essential macroelements (*Ca*, *K*, *Mg*, *Na*, *P*, and *S*) and trace elements (*Co*, *Cu*, *Fe*, *Mn*, and *Se*) were determined in initial and canned fish. About 300 mg of ground sample was put into a digestion flask with 9 mL of 69% nitric acid Hiperpur, 3 mL of H_2_O_2_ (for ultratrace analysis), and 3 mL of Milli-Q water and digested according to the procedure based on EPA 3050B (US-EPA 1996). Quintuplicate samples, plus five blanks and five samples of certified reference material, were digested in an advanced microwave digestion system (ETHOS^TM^ EASY, Milestone, Sorisole, Italy). Sample solutions were transferred to 50 mL flasks. A clean ISO 5 laminar flow cabinet (Cruma 670 FL, Barcelona, Spain) was employed for sample handling.

ICP-MS analysis using an Agilent 7900 instrument (Agilent Technologies, Inc., Santa Clara, CA, USA) was employed for determination of the eleven aforementioned elements. External calibration with element standards traceable to NIST standards was employed. The limits of detection (LD) were calculated by comparison to the standard deviation of the blanks (LD = 3·SD blanks). Procedural blanks always accounted for <1% of element concentrations in the samples. Accuracy of the analytical procedures was ensured using certified reference material DORM-2, prepared by the National Research Council of Canada (Table S1). The reference values of six macroelements in DORM-2 were reported by Engstrom et al. [26] and Millos et al. [27]. The results of the DORM-2 analysis were always within the ranges of certified reference material, except for *Na*, whose contents were slightly lower than certified values (Table S1).

Results were calculated as mg·kg^−1^ dry muscle.

### 2.6. Statistical Analysis

Data (*n* = 5) corresponding to the content of the different macroelements and trace elements were subjected to one-way ANOVA (*p* < 0.05) to analyse the effect of previous frozen storage and coating condition (Statistica version 6.0, 2001; Statsoft Inc., Tulsa, OK, USA) on the content of the different essential elements in the canned product. Differences between initial fish and canned samples were also analysed. In all cases, comparison of means was performed using a least-squares difference (LSD) method.

## 3. Results

### 3.1. Determination of Essential Macroelements 

Concerning the *Na* content, comparison between initial fish and canned fish revealed a marked decrease (*p* < 0.05) in water-coated samples and a strong increase (*p* < 0.05) in brine-canned ones (Table 1); no effect (*p* > 0.05) of the canning process was detected in the case of oil-coated samples. 

Concerning the coating medium effect, canned samples corresponding to water-coating condition showed the lowest (*p* < 0.05) contents, while those coated in brine showed the highest (*p* < 0.05) contents. Additionally, comparison of the three oil-coated samples did not lead to observing differences (*p* > 0.05). The use of the 6-month frozen storage (Group II samples) led to higher average *Na* contents in water-canned and oil-canned samples but lower contents in brine-canned ones; however, differences were not significant (*p* > 0.05).

**Figure 1:**
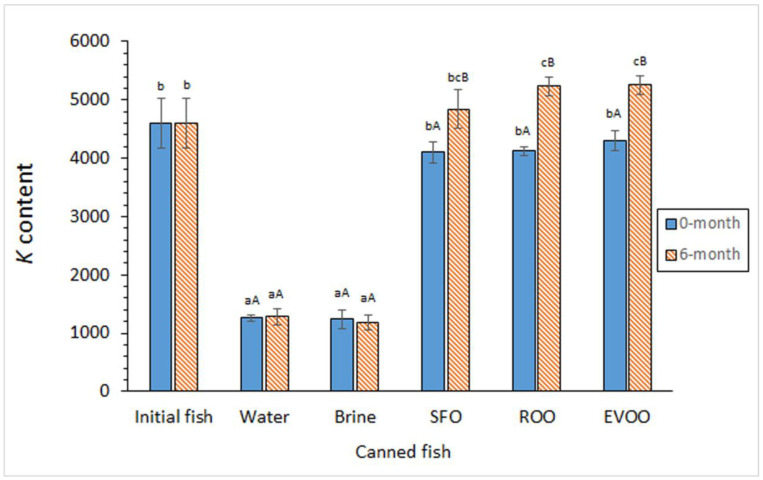
*K* content (mg·kg^−1^ dry muscle) in initial and canned fish muscle subjected to different previous storage and coating conditions. Average values of five replicates (*n* = 5); standard deviations are indicated by bars. For each frozen storage time, different lowercase letters (a–c) indicate significant differences (*p* < 0.05) between initial fish and each of the canned samples corresponding to the different coating conditions. For each coating condition, different capital letters (A, B) indicate significant differences (*p* < 0.05) as a result of the frozen storage period. Abbreviations as expressed in Table S1.

Compared to the initial fish, a marked decrease (*p* < 0.05) in the *K* content was detected in canned fish muscle that was coated in both aqueous media (Figure 1). Fish muscle subjected to oil coating showed lower average contents than the initial fish; however, differences were not found significant (*p* > 0.05). Aqueous-coating samples showed lower (*p* < 0.05) *K* contents than their corresponding oil-coated samples. A *K* content increase (*p* < 0.05) was observed in oil-coated fish muscle as a result of the previous frozen storage; in the case of aqueous-coating samples, no effect (*p* > 0.05) was determined for the previous storage period.

Compared to the initial fish, a remarkable decrease (*p* < 0.05) in *Mg* content was observed in aqueous-coating samples (Table 1); on the contrary, oil-coated ones did not show differences (*p* > 0.05) from initial fish. Among coating conditions, canned fish corresponding to aqueous media showed lower (*p* < 0.05) contents than their corresponding oil-coated samples. Additionally, brine-coated mackerel corresponding to Group II had lower (*p* < 0.05) *Mg* contents than samples corresponding to the water-coated condition.

**Figure 2:**
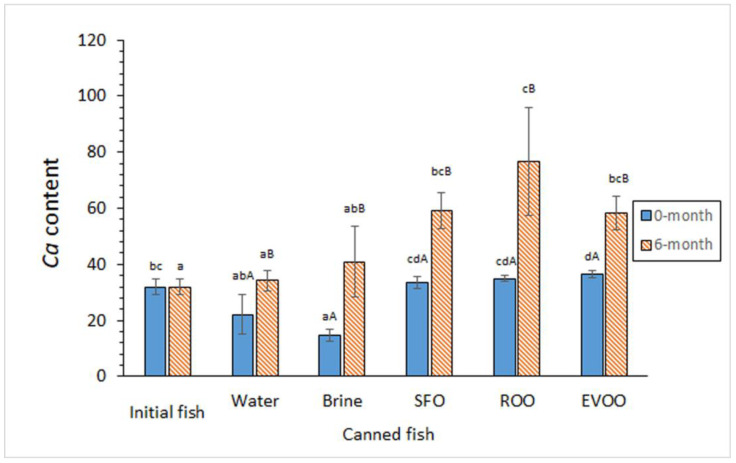
Ca content (mg·kg^−1^ dry muscle) in initial and canned fish muscle subjected to different previous storage and coating conditions. Average values of five replicates (*n* = 5); standard deviations are indicated by bars. For each frozen storage time, different lowercase letters (a–d) indicate significant differences (*p* < 0.05) between initial fish and each of the canned samples corresponding to the different coating conditions. For each coating condition, different capital letters (A, B) indicate significant differences (*p* < 0.05) as a result of the frozen storage period. Abbreviations as expressed in Table S1.

Different tendencies were observed for the *Ca* content when considering fish with or without previous frozen storage (Figure 2). Compared to the initial fish muscle, canned muscle without previous frozen storage showed lower average contents in samples coated in aqueous-coating systems and higher average values in oil-coated samples; differences from initial fish were found significant (*p* < 0.05) in the cases of brine-, ROO-, and EVOO-coated conditions. For canned fish muscle of Group II, higher average *Ca* contents were obtained in all kinds of canned samples when compared to initial fish; differences were only found significant (*p* < 0.05) in oil-coated samples. Higher *Ca* average contents were found in aqueous-coating fish muscle than in their corresponding samples that were coated in oil; differences were found significant (*p* < 0.05) for samples of Group II. A remarkable (*p* < 0.05) *Ca* content increase was detected in all kinds of canned samples as a result of the previous frozen period.

Compared to the initial fish, a marked *P* content decrease (*p* < 0.05) was observed in canned fish coated in both aqueous media (Table 1). In contrast, no effect (*p* > 0.05) was proved when taking into account the oil-canned samples. Oil-coated muscle showed higher (*p* < 0.05) *P* levels than their corresponding aqueous-coating samples. A 6-month previous storage led to a marked decrease (*p* < 0.05) in *P* content in aqueous-coating samples but did not provoke differences (*p* > 0.05) when employing an oil-coated system.

Comparison to initial fish revealed different tendencies for the *S* content in canned fish depending on whether the fish muscle considered was from Group I or II (Table 1). In the case of Group I, no differences (*p* > 0.05) could be outlined by comparison of initial fish and fish muscle canned in both aqueous coating systems; on the contrary, oil-coated fish revealed a remarkable increase (*p* < 0.05). For fish samples of Group II, water-coated samples indicated a relevant decrease (*p* < 0.05) in *S* content when compared to initial fish; however, no differences (*p* > 0.05) were proved for canned ones corresponding to the remaining coating conditions tested. Water-coated samples revealed lower (*p* < 0.05) *S* contents than their corresponding oil-coated samples. Related to the effect of the previous frozen period, average *S* contents were found lower in canned fish muscle corresponding to Group II; differences were found significant (*p* < 0.05) in all cases except for the brine-canned condition.

### 3.2. Determination of Trace Elements

A marked increase (*p* < 0.05) in *Co* content was detected in all kinds of canned samples when compared to initial fish (Table 2). In canned fish corresponding to Group I, higher *Co* contents (*p* < 0.05) were detected in fish coated in SFO and EVOO than in samples corresponding to both aqueous media. For canned fish corresponding to Group II, SFO-coated muscle showed higher (*p* < 0.05) contents than samples corresponding to brine- and ROO-coating conditions. For all kinds of canned fish, no effect (*p* > 0.05) of the previous frozen period could be determined for the *Co* presence. 

Average *Cu* content showed a general increase in canned samples (Table 2). Differences from initial fish were found significant (*p* < 0.05) for oil-coated fish (Groups I and II) and for Group II samples coated in both aqueous media. Higher average *Cu* contents were detected in oil-coated samples than in samples corresponding to aqueous-coating conditions; differences were found significant (*p* < 0.05) in Group II samples. For all coating systems, higher average *Cu* contents were detected in Group II samples when compared to their counterparts from Group I; however, differences were only found significant (*p* < 0.05) for brine-canned samples.

**Figure 3:**
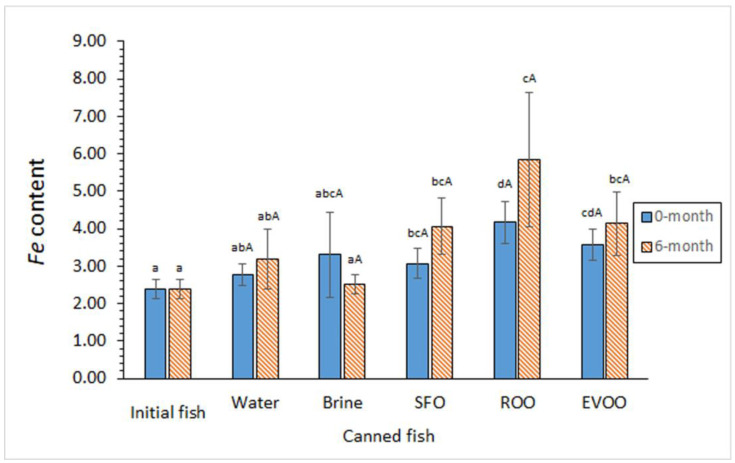
*Fe* content (mg·kg^−1^ dry muscle) in initial and canned fish muscle subjected to different previous storage and coating conditions. Average values of five replicates (*n* = 5); standard deviations are indicated by bars. For each frozen storage time, different lowercase letters (a–d) indicate significant differences (*p* < 0.05) between initial fish and each of the canned samples corresponding to the different coating conditions. (A) No effect (*p* > 0.05) of the frozen storage period was detected. Abbreviations as expressed in Table S1.

Comparison with initial fish revealed an average increase in *Fe* content in all canned samples (Figure 3); differences were found significant (*p* < 0.05) for oil-coated samples for Groups I and II. Comparison among coating systems showed that both olive oil systems led to the highest average *Fe* contents. For samples of Group I, differences between ROO-coated fish and aqueous- and SFO-coating systems were significant (*p* < 0.05). No significant differences (*p* > 0.05) were found as a result of the previous frozen storage; however, oil-coated samples corresponding to Group II led to higher average *Fe* values.

A general average decrease in the *Mn* content was obtained by comparison of initial fish and any kind of canned sample (Table 2). This decrease was found significant (*p* < 0.05) in all kinds of canned samples of Group I. For canned samples corresponding to this group, water-coated fish revealed lower (*p* < 0.05) *Mn* contents than all other canned samples; in the case of canned fish of Group II, ROO-coated fish showed a higher (*p* < 0.05) *Mn* content than aqueous-coating samples. An *Mn* content increase (*p* < 0.05) was observed in water- and ROO-coated samples as a result of the frozen storage period.

The *Se* content showed scarce differences between initial fish and canned samples (Table 2). Thus, only ROO-coated fish muscle (Groups I and II) and brine-coated fish (Group II) led to higher (*p* < 0.05) contents than the initial fish. For samples of Group I, ROO-coated fish muscle showed a higher (*p* < 0.05) *Se* content than their corresponding samples coated in aqueous conditions. Previous frozen storage time led to a *Se* content increase (*p* < 0.05) in the case of brine-coated samples, but no differences (*p* > 0.05) were detected in those corresponding to the remaining coating systems tested.

## 4. Discussion

Previous research provides extensive information on chemical changes related to protein and lipid fractions during seafood canning [16,17,18,19,20,21,22]. On the contrary, data concerning the changes in mineral content related to this technological process can be considered relatively scarce. The present study has shown remarkable changes in the content of most elements in canned fish as a result of the different processing conditions considered. Thus, differences between initial and canned fish corresponding to the different kinds of canned samples have shown different trends (increase, decrease, or no variation) according to the processing condition and the element considered. In order to discuss and justify such variations, a review of the most important events produced by each single processing step or condition applied ought to be carried out.

Concerning the effect of canning (i.e., sterilisation step), constituents from marine species have been shown to be highly sensitive [7,9]. In this sense, oxidation of protein and lipid fractions, heat breakdown, and leaching of constituents into the coating medium have been mentioned [13,28,29]. Remarkably, a detrimental effect on mineral content may be produced as a result of liquor losses from canned fish muscle. Therefore, reduction in mineral contents in fish muscle during the heating process may be related to the protein denaturation and release of elements with the loss of water as free salts, possibly associated with soluble free amino acids and hydrophobic vitamins [12,14,30,31]. This loss ought to be more remarkable when an aqueous medium is employed as coating medium than in the case of using an oil-coating system. On the other hand, denatured proteins are reported to become more reactive and be damaged easily by interacting with other constituents, especially if a heating step such as sterilisation is included. Furthermore, release of prooxidant elements such as non-heme *Fe* from heme-*Fe* complexes may have detrimental consequences for the quality of canned muscle [32,33]. As a result, canned fish may undergo a notable lipid and protein oxidation, leading to breakdown and production of low-molecular-weight molecules susceptible to being lost from the fish tissue [12,13]. Accordingly, a content decrease in constituents such as proteins and lipids in the canned fish muscle would lead to a relative increase in other constituents such as essential minerals. 

According to such considerations, relevant differences between initial fish and canned samples were proved for macroelements and trace elements in the current study. In our study, we observed a decreased content of *Na* and *Mg* (water-coated samples), *P* (water- and brine-coated samples), *S* (water-coated samples of Group II), *K* (water- and brine-coated samples), and *Ca* (brine-coated samples of Group I). On the contrary, a content increase was detected for *Na* (brine-coated samples), *S* (oil-coated samples), *K* (olive-oil-coated samples of Group II), and *Ca* (ROO- and EVOO-coated samples of Group I and oil-coated samples of Group II). As a predominant trend, it can be concluded that a mineral content decrease was observed for aqueous-coating samples, while oil-coated ones showed higher contents than the initial fish.

Previous research accounts for the effect of canning on mineral content in fish muscle. Thus, Seet and Brown [15] proved some loss of minerals (*Ca*, *Na*, *K*, *Mg*, *P*, *Cu*, and *Fe*) from the canned muscle into the water-coating medium in canned tuna (*Thunnus alalunga*). Later on, a loss of protein and water and an increase in lipid content were detected by Castrillón et al. [16] after tuna (*T. alalunga*) canning (sterilisation at 115 °C for 55 or 90 min) by using soy bean oil as coating medium; additionally, a content decrease in some elements (*Mg*, *K*, and *P*) and no effect on others (*Na*, *Ca*, *Cu*, *Fe*, and *Zn*) were detected after steaming. Recently, the canning treatment of brine-canned chub mackerel (*Scomber colias*) led to increased contents of *Na*, *Ca*, *Mn*, *Fe*, *Cu*, *Se*, and *S* but to lower contents of *K*, *Mg*, *Co*, and *P* in canned fish muscle [34]. 

Concerning the frozen storage of seafood, different damage pathways have been described as being responsible for sensory and nutritional losses during such technological processing [17,20,21]. Among them, microstructural changes, protein denaturation, and lipid hydrolysis and oxidation development have proved to be important events for quality loss. As a result of freezing and frozen storage, the fish tissue is reported to become less elastic, more fibrous, harder, and lacking in water-holding capacity [19,35]. This last effect can be especially important for mineral content in frozen seafood and, therefore, in the corresponding canned product. Thus, liquor produced from fish tissue, especially during the needed thawing step, can lead to an important decrease in the mineral content. This element release ought to increase with protein damage and, accordingly, with time and temperature of frozen storage [13,23].

In our study, a strong effect of previous frozen storage on the content of the macroelements tested was observed. Thus, the inclusion of the storage period led to an increased effect in canned fish on *K* (oil-coated samples) and *Ca* (all coating conditions) content and to a decreased content of *P* (aqueous-coating samples) and *S* (water- and oil-coated samples). In the case of trace elements, a content increase in *Cu* and *Se* in brine-canned samples and of *Mn* (water- and ROO-coated samples) was detected as a result of the previous frozen storage period.

Previous research accounting for the effect of frozen storage on the element content in seafood can be considered scarce. Thus, Karl et al. [36] detected a notable reduction in *I* content in different kinds of fish after deep-freezing (−40 °C) and thawing. Pourashouri et al. [37] showed an increase in the non-heme *Fe* content as a result of the *Fe* release from heme-*Fe* complexes during the frozen storage (6 months at −18 °C) of Wels catfish (*Silurus glanis*); this change was explained according to the oxidative cleavage of the porphyrin ring [32,33]. Recently, a previous 6-month frozen storage (−18 °C) led to a general decrease in essential element (*K*, *Mg*, *Ca*, *Mn*, *Fe*, *Se*, *P*, and *S*) content in brine-canned mackerel (*S. colias*) [34]. In a subsequent study, Prego et al. [23] analysed the effect of the previous frozen storage time (–18 °C, up to 15 months) on the content of essential elements in the corresponding brine-canned mackerel (*S. colias*); as a result, an increased frozen storage time led to an increase in *Ca* and *Mn* contents but produced a decrease in *K* content.

Another effect taken into account in the current study is that of the coating medium. The coating medium acts as an extracting medium susceptible to provoking the loss of certain elements in canned muscle. In this sense, the more or less lipophilic or hydrophilic behaviour of molecules in which minerals are integrated would be mandatory in order to foresee important content decreases. It is worth pointing out that interaction of mineral elements with other fish constituents can vary in a wide range and show a great dependence on their chemical characteristics [38,39]. Thus, transition metals (*Fe*, *Cu*, etc.) and non-positive elements (*S*, *P*, etc.) have been reported to be strongly bound to other tissue constituents and lead to a wide number of functional molecules. Contrary, alkali earth (*Mg* and *Ca*) and alkali (*Na* and *K*) elements are known to be present in cellular media as sulphates, organic salts (pyruvates, lactates, citrates), or chlorides. Therefore, a higher loss of alkali and alkali earth elements would be expected to occur when employing an aqueous-coating medium than in the case of using an oil-coated one.

In our study, we observed a marked effect of the coating medium on the content of the macroelements analysed. Thus, aqueous-coating samples showed lower *Mg*, *P*, *K*, and *Ca* contents than their corresponding oil-coated samples. Additionally, a lower *Na* and *S* content was observed in water-coated samples than in those coated in any of the three oils tested. In the case of trace elements, a lower *Co* content was observed in brine-coated samples than in SFO-coated ones; additionally, lower *Cu* contents in brine-coated samples than in their corresponding coated ones in SFO and EVOO were obtained. Water-coated samples showed lower *Mn* contents than fish muscle canned in ROO or EVOO media. Finally, aqueous coating led to lower *Se* (Group I) and *Fe* contents than ROO-coated samples. Because no differences in macroelement and trace element contents have been detected between ROO- and EVOO-coated samples, it is concluded that the presence of natural antioxidant compounds in EVOO (i.e., polyphenol compounds) [17,40] has not led to a different trend in element composition.

Considering the different effects that each condition or single processing step may have on the mineral composition of the current canned mackerel, two opposite effects can be signalled related to changes in the canned seafood. On one hand, each of the different processing steps or conditions applied (freezing, frozen storage, thawing, coating, and sterilisation), especially the last one, would lead to oxidation breakdown, denaturalisation, and partial loss of the main constituents (proteins and lipids, especially) [9,13,19]. As a result, a relative increase in other constituent content such as macroelements and trace elements would be expected to be produced. On the other hand, modifications of constituents, breakdown of binding of minerals to other tissue constituents, and liquor losses from the muscle would lead to a partial loss of minerals into the coating medium [16,30,31]. The significance of this effect would depend on the kind of binding to other tissue constituents and the more or less lipophilic or hydrophilic nature of molecules in which minerals are integrated [39,40]. Elements whose linkage to other constituents is easily lost during any of the processing steps would lead to a more relevant decrease content. On the contrary, those whose linkage to other tissue constituents is not modified during processing would not be likely to be lost and lead to a relative content increase according to the content decrease in other constituents.

Great attention is being accorded currently to the *Na/K* ratio value in human diet. Yang et al. [41] found a reduced risk of cardiovascular diseases if a *Na/K* ratio lower than 1.0 value was present. Remarkably, *Na/K* ratios in the current study were found below 0.7 value in all kinds of canned samples except for those that included brine as coating medium. In that case, higher ratio values than 2 were found, this value being explained on the basis of the great presence of *NaCl* in the coating medium.

## 5. Conclusions

The results show that previous frozen storage led to an increased (*p* < 0.05) content in canned fish of *K* (oil-coated samples) and *Ca* (all coating conditions) and to a decreased (*p* < 0.05) content of *P* (aqueous-coating samples) and *S* (water- and oil-coated samples). For trace elements, a content increase (*p* < 0.05) in *Cu* and *Se* (brine-canned samples) and *Mn* (water- and refined-olive-oil-coated samples) was detected in canned fish of Group II. Concerning the coating effect, aqueous-coating samples showed lower (*p* < 0.05) *Mg, P, S, K,* and *Ca* contents than their corresponding oil-coated samples. For trace elements, lower average contents were detected for *Co*, *Cu*, *Mn*, *Se*, and *Fe* in aqueous-coating samples when compared to those coated in oily media.

## Figures and Tables

**Table 1:** Essential macroelement content (mg·kg^−1^ dry muscle) * in initial and canned fish muscle subjected to different previous storage and coating conditions **.

Element	Previous Storage Time (Months)	Initial Fish	Canned Fish (Coating Condition)				
			**Water**	**Brine**	**SFO**	**ROO**	**EVOO**
*Na*	0	244 ± 29 b	83 ± 4 aA	3740 ± 330 cA	276 ± 20 bA	287 ± 35 bA	285 ± 15 bA
	6	244 ± 29 b	90 ± 8 aA	3580 ± 190 cA	318 ± 30 bA	303 ± 28 bA	311 ± 20 bA
*Mg*	0	348 ± 26 b	128 ± 2 aA	119 ± 16 aA	320 ± 19 bA	314 ± 11 bA	323 ± 10 bA
	6	348 ± 26 c	132 ± 11 bA	101 ± 9 aA	310 ± 23 cA	316 ± 20 cA	324 ± 17 cA
*P*	0	2840 ± 220 b	1160 ± 40 aB	1110 ± 160 aB	2680 ± 90 bA	2710 ± 20 bA	2700 ± 100 bA
	6	2840 ± 220 c	588 ± 73 aA	827 ± 32 bA	2500 ± 140 cA	2830 ± 180 cA	2730 ± 110 cA
*S*	0	2450 ± 210 a	2660 ± 90 aB	2660 ± 190 aA	3510 ± 260 bB	3290 ± 20 bB	3340 ± 120 bB
	6	2450 ± 210 b	1430 ± 130 aA	2340 ± 130 bA	2220 ± 150 bA	2340 ± 150 bA	2420 ± 90 bA

* Average values ± standard deviations (*n* = 5). ** For each frozen storage time, different lowercase letters (a–c) indicate significant differences (*p* < 0.05) as a result of the coating condition. For each coating condition, different capital letters (A, B) indicate significant differences (*p* < 0.05) as a result of the frozen storage period. Abbreviations employed: SFO (sunflower oil), ROO (refined olive oil), and EVOO (extra-virgin olive oil).

**Table 2:** Essential trace element content (mg·kg^−1^ dry muscle) * in initial and canned fish muscle subjected to different previous storage and coating conditions **.

Element	Previous Storage Time (Months)	Initial Fish	Canned Fish (Coating Condition)				
			**Water**	**Brine**	**SFO**	**ROO**	**EVOO**
*Co*	0	1.36 ± 0.28 a	4.96 ± 0.90 bA	4.85 ± 0.80 bA	8.45 ± 1.70 cA	6.76 ± 1.08 bcA	8.12 ± 1.04 cA
	6	1.36 ± 0.28 a	6.95 ± 1.25 bcA	5.44 ± 0.59 bA	7.75 ± 0.36 cA	6.49 ± 0.64 bA	6.59 ± 1.76 bcA
*Cu*	0	0.41 ± 0.13 a	0.66 ± 0.23 abA	0.48 ± 0.13 aA	1.05 ± 0.06 cA	0.95 ± 0.16 bcA	1.25 ± 0.19 cA
	6	0.41 ± 0.13 a	0.68 ± 0.06 bA	0.78 ± 0.09 bB	1.22 ± 0.26 cA	1.13 ± 0.17 cA	1.32 ± 0.25 cA
*Mn*	0	0.064 ± 0.008 c	0.012 ± 0.004 aA	0.031 ± 0.017 bA	0.040 ± 0.009 bA	0.030 ± 0.005 bA	0.032 ± 0.005 bA
	6	0.064 ± 0.008 c	0.030 ± 0.008 aB	0.031 ± 0.009 aA	0.038 ± 0.009 abA	0.051 ± 0.010 bcB	0.043 ± 0.008 abA
*Se*	0	0.318 ± 0.030 a	0.337 ± 0.025 aA	0.298 ± 0.053 aA	0.371 ± 0.055 abA	0.416 ± 0.055 bA	0.379 ± 0.029 abA
	6	0.318 ± 0.030 a	0.304 ± 0.137 abA	0.411 ± 0.055 bB	0.472 ± 0.204 abA	0.576 ± 0.187 bA	0.401 ± 0.084 abA

* Average values ± standard deviations (*n* = 5) expressed as mg·kg^−1^ dry muscle, except for *Co* (μg·kg^−1^ dry muscle). ** For each frozen storage time, different lowercase letters (a–c) indicate significant differences (*p* < 0.05) as a result of the coating condition. For each coating condition, different capital letters (A, B) indicate significant differences (*p* < 0.05) as a result of the frozen storage period. Abbreviations as expressed in Table S1.

## Data Availability

All related data and methods are presented in this paper. Additional inquiries should be addressed to the corresponding author.

## Supplementary Materials

The following supporting information can be downloaded at: https://www.mdpi.com/article/10.3390/foods12122289/s1, Table S1: Accuracy control of the analytical procedure for the determination of macroelements and trace elements in initial and canned samples corresponding to Groups I and II*. Certified reference material DORM-2 (National Research Council of Canada, NRCC) was employed for trace elements. In the case of macroelements, contents come from Engstrom et al. [26] and Millos et al. [27].

## References

[1] Fraga C.G. (2005). Relevance, essentiality and toxicity of trace elements in human health. Mol. Asp. Med..

[2] Roos N., Wahab N.A., Chamnan C., Thilsted S.H. (2007). The role of fish in foodbased strategies to combat vitamin A and mineral deficiencies in developing countries. J. Nutr..

[3] Oehlenschläger J., Nollet L., Toldrá F. (2010). Minerals and trace elements. Handbook of Seafood and Seafood Products Analysis.

[4] Martínez-Valverde I., Periago M.J., Santaella M., Ros G. (2000). The content and nutritional significance of minerals on fish flesh in the presence and absence of bone. Food Chem..

[5] Noël L., Chafey C., Testu C., Pinte J., Velge P., Guérin T. (2011). Contamination levels of lead, cadmium and mercury in imported and domestic lobsters and large crab species consumed in France: Differences between white and brown meat. J. Food Comp. Anal..

[6] Vieira E.F., Soares C., Machado S., Oliva-Teles M.T., Correia M., Ramalhosa M.J., Carvalho A., Domingues V.F., Antunes F., Morais S. (2020). Development of new canned Chub mackerel products incorporating edible seaweeds- Influence on the minerals and trace elements composition. Molecules.

[7] Veiga A., Martínez E., Ojea G., Caride A., Cabado A.G., Vieites J.M. (2008). Principles of thermal processing in canned seafood. Quality Parameters in Canned Seafoods.

[8] Tokur B., Korkmaz K., Özoğul Y. (2020). Novel thermal sterilisation technologies in seafood processing. Innovative Technologies in Seafood Processing.

[9] Lukoshkina M., Odoeva G. (2003). Kinetics of chemical reactions for prediction of quality of canned fish during storage. App. Biochem. Microb..

[10] Pitarch J.L., Vilas C., de Prada C., Palacín C.G., Alonso A.A. (2021). Optimal operation of thermal processing of canned tuna under product variability. J. Food Eng..

[11] FAO Inform (2021). Fishery and Aquaculture Statistics. Commodities, Yearbook 2019.

[12] Pérez-Martín R.I., Franco J.M., Aubourg S., Gallardo J.M. (1988). Changes in free amino acids content in albacore (*Thunnus alalunga*) muscle during thermal processing. Z. Lebensm. Unters. Forsch..

[13] Aubourg S.P. (2001). Review: Loss of quality during the manufacture of canned fish products. Food Sci. Technol. Int..

[14] Mújica-Paz H., Valdez-Fragoso A., Samson C.T., Welti-Chanes J., Torres J.A. (2011). High-pressure processing technologies for the pasteurization and sterilization of foods. Food Bioprocess Technol..

[15] Seet S., Brown D. (1983). Nutritional quality of raw, precooked and canned albacore tuna (*Thunnus alalunga*). J. Food Sci..

[16] Castrillón A.M., Navarro M.P., García-Arias M.T. (1996). Tuna protein nutritional quality changes after canning. J. Food Sci..

[17] Aubourg S.P. (2023). Enhancement of lipid stability and acceptability of canned seafood by addition of natural antioxidant compounds to the packing medium. A review. Antioxidants.

[18] Aubourg S.P., Rey-Mansilla M., Sotelo C. (1999). Differential lipid damage in various muscle zones of frozen hake (*Merluccius merluccius*). Z. Lebensm. Unters. Forsch..

[19] Sikorski Z., Kolakowski E., Haard N., Simpson B. (2000). Endogenous enzyme activity and seafood quality: Influence of chilling, freezing, and other environmental factors. Seafood Enzymes.

[20] Aubourg S.P., Piñeiro C., González M.J. (2004). Quality loss related to rancidity development during frozen storage of horse mackerel (*Trachurus trachurus*). J. Amer. Oil Chem. Soc..

[21] Kolakowska A., Sikorski Z., Kolakowska A. (2003). Lipid Oxidation in Food Systems. Chemical and Functional Properties of Food Lipids.

[22] Naseri M., Rezaei M. (2012). Lipid changes during long-term storage of canned sprat. J. Aquat. Food Prod. Technol..

[23] Prego R., Martínez B., Cobelo-García A., Aubourg S.P. (2021). Effect of high-pressure processing and frozen storage prior to canning on the content of essential and toxic elements in mackerel. Food Bioprocess Technol..

[24] Ruiz-Roso B., Cuesta I., Pérez M., Borrego E., Pérez-Olleros L., Vare G. (1998). Lipid composition and palatability of canned sardines. Influence of the canning process and storage in olive oil for five years. J. Sci. Food Agric..

[25] AOAC (1990). Official Methods for Analysis of the Association of Analytical Chemistry.

[26] Engström E., Stenberg A., Senioukh S., Edelbro R., Baxter D.C., Rodushkin I. (2004). Multi-elemental characterization of soft biological tissues by inductively coupled plasma–sector field mass spectrometry. Anal. Chim. Acta.

[27] Millos J., Costas-Rodríguez M., Lavilla I., Bendicho C. (2009). Multiple small volume microwave-assisted digestions using conventional equipment for multielemental analysis of human breast biopsies by inductively coupled plasma optimal emission spectrometry. Talanta.

[28] Gokoglu N., Yerlikaya P., Cengiz E. (2004). Effects of cooking methods on the proximate composition and mineral contents of rainbow trout (*Oncorhynchus mykiss*). Food Chem..

[29] Mierke-Klemeyer S., Larsen R., Oehlenschläger J., Maehre H., Elvevoll E.O., Bandarra N.M., Parreira R., Andrade A.M., Nunes M.L., Schram E. (2008). Retention of health-related beneficial components during household preparation of selenium-enriched African catfish (*Clarias gariepinus*) fillets. Eur. Food Res. Technol..

[30] García-Arias M.T., Sánchez-Muniz F.J., Castrillón A.M., Navarro M.P. (1994). White tuna canning, total fat, and fatty acid changes during processing and storage. J. Food Comp. Anal..

[31] Ganjavi M., Ezzatpanah H., Givianrad M.H., Shams A. (2010). Effect of canned tuna fish processing steps on lead and cadmium contents of Iranian tuna fish. Food Chem..

[32] Buchowski M.S., Mahoney A.W., Carpenter C.E., Cornforth D.P. (1988). Heating and the distribution of total and heme iron between meat and broth. J. Food Sci..

[33] Turhan S., Sule Ustun N., Bogachan Altunkaynak T. (2004). Effect of cooking methods on total and heme iron contents of anchovy (*Engrauslis encrasicholus*). Food Chem..

[34] Prego R., Vázquez M., Cobelo-García A., Aubourg S.P. (2020). Macroelements and trace elements content in brine-canned mackerel (*Scomber colias*) subjected to high-pressure processing and frozen storage. Foods.

[35] Suvanich V., Jahncke M.L., Marshall D.L. (2000). Changes in selected chemical quality characteristics of Channel catfish frame mince during chill and frozen storage. J. Food Sci..

[36] Karl H., Basak S., Ziebell S., Quast P. (2005). Changes in the iodine content in fish during household preparation and smoking. Deut. Lebensm. Rundsch..

[37] Pourashouri P., Shabanpour B., Aubourg S.P., Daghigh Rohi J., Shabani A. (2009). An investigation of rancidity inhibition during frozen storage of Wels catfish (*Silurus glanis*) fillets by previous ascorbic and citric acid treatment. Int. J. Food Sci. Technol..

[38] Piclet G. (1987). Le poisson aliment. Composition—intérêt nutritionnel. Cah. Nutr. Diét..

[39] Gordon D.T. (1988). Minerals in seafoods: Their bioavailability and interactions. Food Technol..

[40] Medina I., Sacchi R., Biondi L., Aubourg S.P., Paolillo L. (1998). Effect of packing media on the oxidation of canned tuna lipids. Antioxidant effectiveness of extra virgin olive oil. J. Agric. Food Chem..

[41] Yang Q., Liu T., Kuklina E.V., Flanders W.D., Hong Y., Gillespie C., Chang M., Gwinn M., Dowling N., Khoury M. (2011). Sodium and potassium intake and mortality among US adults: Prospective data from the Third National Health and Nutrition Examination Survey. Arch. Int. Med..

