# Supplementary material for: Effect of Previous Frozen Storage and Coating Medium on the Essential Macroelement and Trace Element Content of Canned Mackerel
Source: Foods. 2023 Jun 6;12(12):2289. doi: 10.3390/foods12122289 (PMC10296788; doi:10.3390/foods12122289)
Supplement: Supplementary file 1 [file foods-12-02289-s001.zip › foods-2391843-supplementary.pdf]

TABLE S1

Accuracy control of the analytical procedure for the determination of macroelements and trace elements in initial and canned samples corresponding to Groups I and II*. Certified reference material DORM-2 (National Research Council of Canada, NRCC) was employed for trace elements. In the case of macroelements, contents come from Engstrom et al. [26] and Millos et al. [27].

Element	Certified	Initial and Group I samples	Group II samples
**MACROELEMENTS**	g·kg^{-1}	g·kg^{-1}	g·kg^{-1}
Ca [26]	0.620 ± 0.050		0.650 ± 0.045
Ca [27]	0.485 ± 0.043	0.465 ± 0.015	
K [26]	18.9 ± 1.1		18.10 ± 0.29
K [27]	14.42 ± 0.29	13.30 ± 0.35	
Mg [26]	1.050 ± 0.050		0.970 ± 0.020
Mg [27]	1.024 ± 0.085	0.980 ± 0.025	
Na [26]	5.06 ± 0.07	4.45 ± 0.12	4.75 ± 0.10
Na [27]	no data		
P [26]	9.92 ± 0.14		10.00 ± 0.22
P [27]	10.11 ± 0.23	9.35 ± 0.20	
S [26]	8.92 ± 0.49		8.90 ± 0.20
S [27]	7.96 ± 0.27	8.39 ± 0.30	
**TRACE ELEMENTS**	mg·kg^{-1}	mg·kg^{-1}	mg·kg^{-1}
Co	0.18 ± 0.03	0.17 ± 0.01	0.16 ± 0.01
Cu	2.34 ± 0.16	2.17 ± 0.19	2.19 ± 0.14
Fe	142 ± 10	141 ± 7	125 ± 5
Mn	3.66 ± 0.34	3.47 ± 0.17	3.36 ± 0.13
Se	1.40 ± 0.09	1.33 ± 0.06	1.41 ± 0.19

* Data expressed as average content ± standard deviation (n = 5).
